# Gaze Bias in Preference Judgments by Younger and Older Adults

**DOI:** 10.3389/fnagi.2017.00285

**Published:** 2017-08-25

**Authors:** Toshiki Saito, Rui Nouchi, Hikari Kinjo, Ryuta Kawashima

**Affiliations:** ^1^Division of Advanced Brain Science, Institute of Development, Aging and Cancer, Tohoku University Sendai, Japan; ^2^Creative Interdisciplinary Research Division, Frontier Research Institute for Interdisciplinary Science (FRIS), Tohoku University Sendai, Japan; ^3^Human and Social Response Research Division, International Research Institute of Disaster Science, Tohoku University Sendai, Japan; ^4^Faculty of Psychology, Department of Psychology, Meiji Gakuin University Tokyo, Japan

**Keywords:** eye movements, gaze bias effect, preference, decision-making, aging

## Abstract

Individuals’ gaze behavior reflects the choice they will ultimately make. For example, people confronting a choice among multiple stimuli tend to look longer at stimuli that are subsequently chosen than at other stimuli. This tendency, called *the gaze bias effect*, is a key aspect of visual decision-making. Nevertheless, no study has examined the generality of the gaze bias effect in older adults. Here, we used a two-alternative forced-choice task (2AFC) to compare the gaze behavior reflective of different stages of decision processes demonstrated by younger and older adults. Participants who had viewed two faces were instructed to choose the one that they liked/disliked or the one that they judged to be more/less similar to their own face. Their eye movements were tracked while they chose. The results show that the gaze bias effect occurred during the remaining time in both age groups irrespective of the decision type. However, no gaze bias effect was observed for the preference judgment during the first dwell time. Our study demonstrated that the gaze bias during the remaining time occurred regardless of decision-making task and age. Further study using diverse participants, such as clinic patients or infants, may help to generalize the gaze bias effect and to elucidate the mechanisms underlying the gaze bias.

## Introduction

Our gaze has a strong effect on our decision-making processes. Shimojo et al. (2003) found that participants’ gaze shifted gradually toward a chosen stimulus before their decision to select that stimulus was implemented. Their results demonstrated that the gaze biases were specifically more pronounced in preference decisions than under the control conditions. They called this profound gaze bias effect in preference decisions *the gaze cascade effect*. Moreover, longer exposure to the stimulus increased the likelihood of choosing it in the preference decision. Consequently, Shimojo et al. (2003) concluded that the profound gaze bias effect observed in the preference task differs from the general gaze bias in non-preference tasks, such as response-related behavior, because attention toward an object, such as looking at a stimulus and preferring it, are intrinsically and mutually linked (Simion and Shimojo, 2006, 2007).

However, some more recent studies have expanded the gaze bias effect to other decision judgments. In some studies, similar data patterns were found under both the preference and the non-preference decision conditions (Glaholt and Reingold, 2009a,b; Nittono and Wada, 2009; Schotter et al., 2010; Mitsuda and Glaholt, 2014; Saito et al., 2015). Glaholt and Reingold (2009a) reported that selective encoding drives the gaze bias in decision-making tasks in general. Selective encoding involves more processing for the decision-relevant features of the stimulus. For example, when people choose an option that they like/dislike, they allocate more attention to the pleasant/unpleasant features of the option. Schotter et al. (2010) suggested that gaze data be divided into two parts based on time courses (first dwell time and remaining time) and that each part reflect a different stage of the decision-making process. First dwell time reflects the encoding stage of the decision-making process, whereas remaining time reflects a post-encoding stage. If selective encoding modulates the gaze bias effect, then the size and direction of the gaze bias effect would differ during the first dwell time but not during the remaining time because the remaining time would be more likely to reflect the response-related aspects of the decision process (i.e., the tendency to look at an option while making a response). In the like decision, the gaze bias would become more profound because selective encoding is consistent with a liking effect in which there is a tendency to look longer at a liked option (Fantz, 1964). However, in the dislike decision, the gaze bias would weaken because selective encoding is inconsistent with the liking effect. Accordingly, gaze bias does not occur in a dislike decision. Indeed, Schotter et al. (2010) reported the gaze bias in the first dwell time in like judgments but not in dislike judgments. However, they observed the gaze bias in both tasks in the remaining time. Supporting these results, many previous studies have described that gaze bias during the remaining time occurs in any judgment task (Glaholt and Reingold, 2009a,b; Schotter et al., 2010; Saito et al., 2015). For instance, Saito et al. (2015) reported that when participants chose the face that they regarded as older than the other, a profound gaze bias effect also occurred in response to both the older face and the like judgments. Based on these findings, Schotter et al. (2010) concluded that the gaze bias effect in the remaining time would occur under any kind of decision-making condition.

Previous studies examined only young cohorts, such as undergraduate students and/or graduate students. To assess the generality of the gaze bias effect, we must determine whether the gaze bias effect also occurs in older adults. The cognitive performance and behaviors of older and younger adults differ. One common factor is the decline in the speed of performance in older adults (e.g., Ratcliff et al., 2001). Even on a simple detection task, older adults exhibit slower performance. Another factor is age-related differences in the processing of emotional information. Older adults tend to focus on positive emotional stimuli rather than on neutral and negative emotional stimuli (Carstensen and Mikels, 2005). These positive effects on attention and memory are supported by mounting evidence in the literature (Reed et al., 2014). These findings suggest the importance of investigating whether gaze bias in decision-making-related emotions, such as like or dislike judgments, occurs in older adults.

This study was conducted to generalize the gaze bias effect in older adults based on findings reported by Schotter et al. (2010). This study was designed to test the generality of the gaze bias across age groups. Therefore, we used typical decision-making conditions (two-alternative forced-choice task; 2AFC) and compared the gaze behaviors of younger adults to those of older adults. We hypothesized that the gaze bias would be apparent in younger and older adults. Second, if the extent of the gaze bias at the encoding stage were affected by the liking effect (Schotter et al., 2010), then the extent of the gaze bias in older adults would expected to be greater than that of younger adults during like decisions. Additionally, the degree of the gaze bias in older adults would be expected to be less than that of younger adults in dislike decisions because older adults have a strong tendency to prefer positive stimuli (Reed et al., 2014).

## Materials and Methods

### Participants

Eighteen young adults (15 females, aged 20–23 years, *M* = 20.8 years) and 20 older adults (10 females, aged 65–74, *M* = 69.7 years) participated. The younger adults were students at Meiji Gakuin University. The older adults were recruited from a local community job center where they were registered for temporary jobs. All participants had normal or corrected-to-normal vision. The younger adults received small gifts whereas the older adults received about $20 for their participation. Data from one younger and one older male participant and two younger female participants were excluded from analyses because of mechanical failure.

The older and younger adults did not differ with regard to years of education (*t*_(36)_ = 1.55, *n.s.*, *d* = 0.50). No older adult reported a diagnosis of dementia or a past or present neuropsychological disorder. The experiment was conducted in accordance with the ethics policy of Meiji Gakuin University and the principles outlined in the Declaration of Helsinki. Written informed consent was received from each participant.

### Apparatus

Eye movements were recorded using a screen-based eye tracker (60 Hz) integrated into a 17-″ TFT monitor (T60; Tobii Technology., Stockholm, Sweden). Stimuli were presented on a monitor with a 1280 × 1024 pixel resolution. The distance between each participant and the display was about 60 cm.

### Stimuli

**Figure 1:**
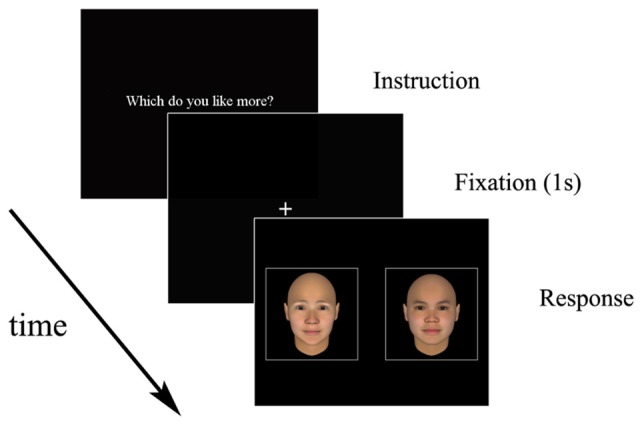
Example of stimuli presented to participants.

In total, 200 three-dimensional human faces generated using software (FaceGen Modeller 3.5^1^) were used as stimuli. Following the procedure used to assess the attractiveness of the stimulus faces reported by Shimojo et al. (2003), we asked 16 observers who differed from the participants to rate all faces from 1 (very unattractive) to 7 (very attractive). The age range of the raters was 20–22 years (*M* = 20.88, *SD* = 0.70). The average ratings of the faces in a pair were matched so that the difference in the average rating in each pair was <0.20 points. The average rating for all faces was 2.87 (*SD* = 0.70). The faces in a pair were matched for gender and race and showed a natural facial expression. The face pairs were presented side-by-side on a black background (Figure 1).

### Procedure

The participants were tested individually in a quiet room. They were seated in front of the monitor at a distance of approximately 60 cm. Room lighting was provided by overhead fixed fluorescent lamps. After obtaining informed consent from each participant, we asked the participants to perform three training trials to practice and to ensure that they understood the task. They were instructed to try their best not to move their head during the task.

After a fixation point was presented at the center of the screen for 1 s, the paired faces were presented side-by-side on the screen with a visual angle of 10.0° each. The participants were instructed to inspect the faces freely and to press the button corresponding to the respective face. Participants performed the following four decision tasks in a pseudo-randomized order: (1) the like task, in which they were asked to choose the more attractive face; (2) the dislike task, in which they were asked to choose the less attractive face; (3) a similar task, in which they chose a face similar to their own; and (4) a dissimilar task, in which they chose a face dissimilar to their own. Each task consisted of 23 trials. The order of trials was randomized across participants. The eye tracker was calibrated before each task to ensure that it was accurately recording the eye positions. Participants performed four tasks (like, dislike, similar and dissimilar) in a single session of approximately 30 min per day. Each session occurred between 9 a.m. and 5 p.m.

### Statistical Analysis

This study was designed to: (1) determine whether the gaze cascade effect occurs in older adults; and (2) explore the effect of aging on gaze behavior during decision-making. Trials with response times ±3 SDs from each participant’s mean were excluded as outliers. Consequently, 0.80% of trials were excluded.

We performed corrected mixed-measures analysis of variance (ANOVA) to analyze reaction time. Our design was 2 (age group: younger and older adult) × 4 (decision type: like, dislike, similar and dissimilar). Additionally, the response time data were log-transformed before statistical testing.

We conducted a gaze likelihood curve analysis (Shimojo et al., 2003) to analyze the gaze behavior data. The gaze likelihood curve shows the likelihood that the chosen face was inspected at each sampling point. We assigned a true value (1) to every sampling point when a participant looked at the chosen face, and a false value (0) when a participant looked at the unchosen face. When participants did not look at either face, we assigned “not-a-number”. We analyzed the gaze likelihood at the time of decision (the last gaze likelihood) in a 2 (age group) × 4 (decision type) mixed-measures ANOVA. The Kolmogorov–Smirnov test was used to examine the differences in the likelihood curves between age groups.

To assess the gaze data in detail, we conducted a dwell duration analysis (Schotter et al., 2010). We divided the gaze data into the first dwell time and the remaining time. The first dwell time was defined as the sum of all fixations on an item before leaving it. This measure captured the encoding stage of the decision process because it constituted the first time a stimulus was encountered. The remaining time was defined as the sum of fixations on a stimulus throughout one trial minus the first dwell time. The remaining time was assumed to reflect post encoding and the decision–response process. We applied the Hyunh–Feldt corrected mixed-measures ANOVA to analyze first dwell time and the remaining time. Our design was 2 (age group) × 4 (decision type) × 2 (choice: chosen, unchosen). The age group was the between-subjects factor.

We conducted a *post hoc* analysis using the modified sequentially rejective Bonferroni method for all ANOVAs. A *p*-value < 0.05 with the Bonferroni correction was considered significant.

## Results

### Response Time

The analysis revealed a significant main effect of decision type (*F*_(3,96)_ = 3.41, *p* = 0.02, $\eta_{\text{p}}^{2}$ = 0.10; Table 1). *Post hoc* comparisons indicated that participants responded faster under the dislike condition than under the similar and dissimilar conditions (*t*s_(32)_ = 2.92 and 2.79, *p*s = 0.006 and 0.009). No significant main effect was found for age (*F*_(1,32)_ = 1.21, *n.s*., $\eta_{\text{p}}^{2}$ = 0.04). However, we found a significant interaction for age × decision type (*F*_(3,96)_ = 5.26, *p* = 0.002, $\eta_{\text{p}}^{2}$ = 0.14). The simple main effects tests demonstrated that younger adults responded significantly faster than the older adults to the dislike decision (*F*_(1,32)_ = 6.99, *p* = 0.01, $\eta_{\text{p}}^{2}$ = 0.18). Additionally, an effect of decision type was found in younger adults (*F*_(3,42)_ = 5.67, *p* = 0.002, $\eta_{\text{p}}^{2}$ = 0.29). A multiple comparison analysis revealed that the response time for the dislike decision was shorter than those for the similar and dissimilar decisions among younger adults (*t*s_(14)_ = 4.65 and 3.65, *p*s = 0.001 and 0.003).

**Table 1:** Average reaction times (s).

Decision types	Older (s)	Young (s)	Significance
Like	3.72 (0.34)	3.44 (0.60)	*n.s.*
Dislike	3.58 (0.23)	2.71 (0.30)	*p* = 0.01
Similar	3.58 (0.30)	3.59 (0.41)	*n.s.*
Dissimilar	3.47 (0.22)	3.71 (0.55)	*n.s.*

*Note: Standard deviations are given in parentheses*.

### Gaze Behavior

#### Gaze Likelihood Curve

**Figure 2:**
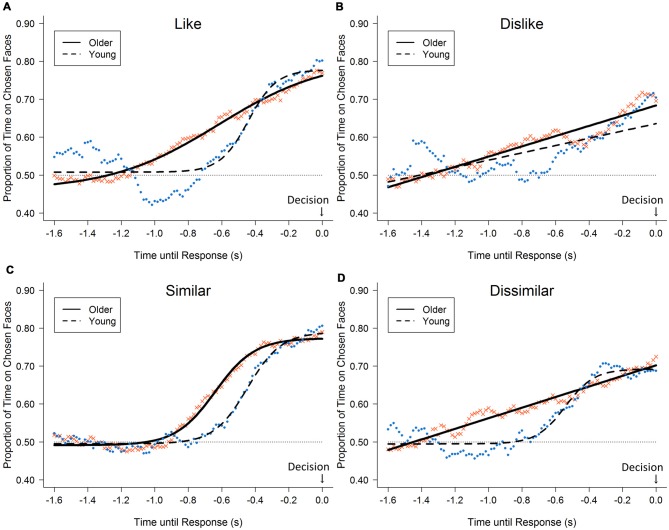
Likelihood of gazing at the chosen face for 1.6 s before response: black circles represent the average across young adults; crosses represent older adults; lines represent fitted curves separately for younger adults (dashed lines) and older adults (solid lines). Each panel shows results for each of the four decision tasks: **(A)** like, **(B)** dislike, **(C)** similar, and **(D)** dissimilar tasks; horizontal dotted line represents the chance level (50%).

The gaze likelihood curves show a progressive bias toward the chosen faces under all conditions (Figure 2). The curves rose more steeply, especially under the like and similar conditions (younger, up to 80% and 81%; older, up to 77% and 79%, respectively). The curves for the dislike and dissimilar conditions rose less steeply (younger, up to 70% and 69%; older, up to 70% and 72%, respectively). The analysis of the gaze likelihood at the time of the button press revealed a significant main effect of decision type (*F*_(3,96)_ = 8.50, *p* < 0.001, $\eta_{\text{p}}^{2}$ = 0.21). *Post hoc* analyses showed that the like and similar decisions were associated with a greater likelihood than the dislike and dissimilar decisions that, at the time of the button press, gaze would be directed toward the chosen stimuli. However, no significant effect of age group and no significant interaction were found (*F*_(1,32)_ = 0.01, *n.s*., $\eta_{\text{p}}^{2}$ < 0.001; *F*_(3,96)_ = 0.71, *n.s.*, $\eta_{\text{p}}^{2}$ = 0.02).

A significant difference was found between older and young adults in the analysis of the gaze likelihood curve under the dissimilar condition (*d* = 0.31, *p* < 0.001). Additionally, marginal trends toward significance were found under the like and dislike conditions (*d*s = 0.22, *p*s = 0.084), where the likelihood curves of older adults started to rise earlier than did those of the younger adults. However, no significant difference was found under the similar condition (*d* = 0.16, *n.s.*). We conducted the following dwell-duration analysis to examine these age-related differences.

#### First Dwell Time

**Figure 3:**
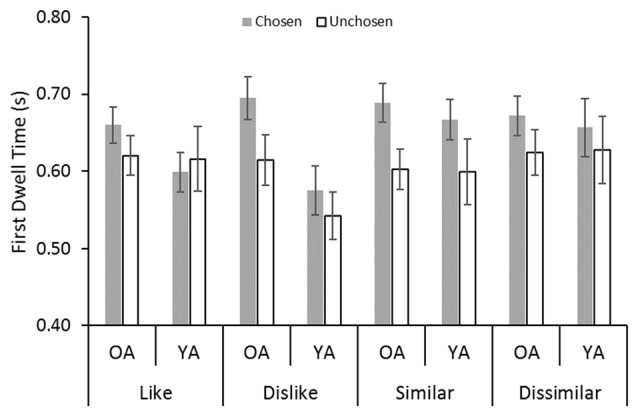
Average dwell duration values in the first dwell time epoch: YA, younger adults; OA, older adult. Error bars represent standard errors.

Figure 3 shows a significant gaze bias effect for the first dwell time: participants spent a longer time looking at a chosen stimulus than at an unchosen stimulus (*F*_(1,32)_ = 14.67, *p* < 0.001, $\eta_{\text{p}}^{2}$ = 0.31). No significant effect of age was found (*F*_(1,32)_ = 0.96, *n*.*s.*, $\eta_{\text{p}}^{2}$ = 0.03). We found an interaction between age and decision type (*F*_(3,96)_ = 3.04, *p* = 0.03, $\eta_{\text{p}}^{2}$ = 0.09). *Post hoc* analyses showed that younger adults looked at the stimuli in the dislike task for a shorter period than did older adults.

Most importantly, a significant interaction was found between decision type and choice (*F*_(2.95,94.42)_ = 2.73, *p* = 0.05, $\eta_{\text{p}}^{2}$ = 0.08). *Post hoc* analyses showed gaze bias effects in the dislike and similar decisions. However, no gaze bias effects were found in the like and dissimilar decisions. These results are inconsistent with the findings of Schotter et al. (2010), which indicated that gaze bias was present for the like decision, but it was absent for the dislike decision.

Appendix 1 in Supplementary Material presents a table showing the average dwell duration values across age groups and conditions.

#### Remaining Time

**Figure 4:**
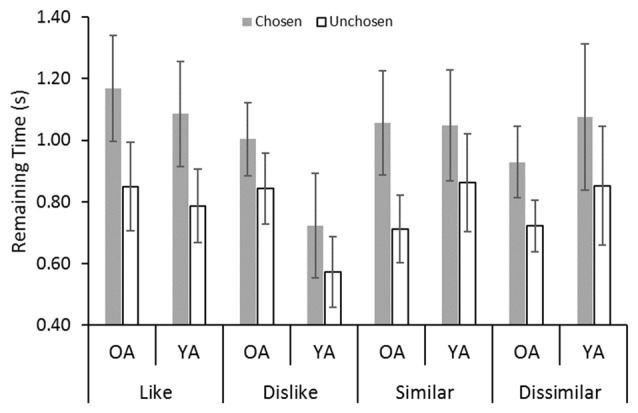
Average dwell duration values in the remaining time epochs: YA, younger adult; OA, older adult. Error bars represent standard errors.

Figure 4 shows that the gaze bias in remaining time was similar to that in the first dwell time. We also found significant gaze bias effects in remaining time (*F*_(1,32)_ = 58.17, *p* < 0.001, $\eta_{\text{p}}^{2}$ = 0.65). No significant effect of age was found (*F*_(1,32)_ = 0.03, *n.s*., $\eta_{\text{p}}^{2}$ < 0.001). We found a significant interaction between decision type and choice (*F*_(3,96)_ = 3.97, *p* = 0.01, $\eta_{\text{p}}^{2}$ = 0.11). The results of the *post hoc* analysis showed that the gaze bias effect was the greatest in the dislike decision.

Little difference was found in the results for the remaining time measures and the first dwell time. In contrast, we observed gaze biases in all decisions in the remaining time analysis, which is consistent with earlier findings (Glaholt and Reingold, 2009a,b; Schotter et al., 2010, 2012; Mitsuda and Glaholt, 2014). These results indicate that the gaze bias is generally observed across ages.

## Discussion

Previous studies have investigated gaze behavior during decision-making but have done so only in younger adults; thus, it remains unclear whether gaze bias in decision-making can be generalized across generations. To address this issue, we compared the behavior of younger and older adults under several types of decision-making conditions during the 2AFC task. Based on previous findings, we developed two main hypotheses. First, that gaze bias is apparent in both younger and older adults. Second, that the extent of the gaze bias of older adults would be greater than that of younger adults in like decisions. Additionally, we expected that the extent of the gaze bias of older adults would be less than that of younger adults for dislike decisions because of the age-related positivity effect (Reed et al., 2014). Our results supported the first, but not the second hypothesis. We discuss these results separately below.

Our results regarding the first hypothesis showed that the gaze bias occurred in both younger and older adults. Gaze bias toward chosen stimuli was observed in both older and younger adults in terms of an increase in the dwell duration and the likelihood of gaze. Consistent with the report by Schotter et al. (2010), gaze bias was present not only in the remaining time but also in the first dwell time. Therefore, we confirmed that the gaze bias did not result from response-related behavior and that the gaze bias can be generalized to older adults.

We expected to observe a stronger interaction between decision type and choice in older adults than in younger adults because of age-related traits, such as the positivity effect. However, we did not find a stronger gaze bias in older adults during a decision task related to preferences. According to Schotter et al. (2010), gaze bias under the preference-related condition is modulated by selective encoding and the liking effect. Their study found an interaction with first dwell time. When their participants chose the liked item, selective encoding worked in concert with the liking effect, and a large gaze bias effect was found. However, when their participants chose the disliked item, no gaze bias effect was found because the liking effect competed with selective encoding. Assuming that the Schotter et al. (2010) explanation is accurate, we expected that older adults would show a stronger gaze bias than younger adults because older adults tend to look at like stimuli longer than younger adults do. Inconsistent with this prediction, the gaze bias in the older adults was not boosted by the positivity effect. One possible explanation for this result was provided by Reed et al. (2014). According to them, the positivity effect is stronger in studies that do not constrain cognitive processing (e.g., free viewing tasks). In this study, we constrained cognitive processing by asking participants to choose between faces. It is possible that the positivity effect was not observed for this reason.

Similar to Schotter et al. (2010), we found an interaction between decision type and choice for first dwell time but not for remaining time. Therefore, we expected that the general gaze bias in the encoding stage, such as that involving selective encoding, would be affected by some additive effects. However, inconsistent with Schotter et al. (2010), for reasons that remain unclear, the gaze bias during first dwell time was observed in the dislike decision but not in the like decision. A possible explanation for this difference is that the participants used different decision strategies because of the attractiveness of the stimuli. Previous studies used a dwell duration analysis (Glaholt and Reingold, 2009b; Schotter et al., 2010) and photographs (e.g., landscapes, portraits, and animals), but we used more and less unattractive human faces (*M* = 2.87, on a 1–7 scale). In the previous study, the unchosen stimuli in the dislike decision would be preferred. However, in this study, the unchosen stimuli in the dislike decision might not be preferred because the stimuli were less attractive. Moreover, our study is the first to use a dwell duration analysis on stimuli consisting of human faces. Perhaps because of these differences, the liking effect toward unchosen stimuli did not work under the dislike condition. Further studies must be undertaken to explore the effect of the attractiveness of stimuli on first dwell time.

This study specifically compared the gaze of older adults with that younger adults according to decision condition. The likelihood curve analysis showed differences among the like, dislike, and dissimilar decisions. In these decisions, the curves of older adults started to rise earlier than did those of the younger adults. A possible explanation for these differences in the likelihood curve analysis is related to general slowness associated with aging. Gaze bias might reflect participants’ tendency to continue fixating on the chosen stimulus after the decision but before the recording of the response. Generally, the motor response becomes slower with age (Ratcliff et al., 2001). Accordingly, the interval between the decision and the recording of the response might be prolonged by the general slowness associated with aging. Indeed, the gaze curves of the older adults rose earlier than those of the younger adults. However, as described by Glaholt and Reingold (2009b), interpreting the gaze likelihood curves is complex because it may reflect either longer time spent dwelling on the chosen stimulus, more frequent dwelling on the chosen item, or a mixture of both. Additionally, as Schotter et al. (2010) reported, gaze likelihood curves provide a visual representation of the probability of looking at the chosen item during the response, but they are not informative beyond that. Therefore, these differences might not be an important feature of our results.

This study had several limitations. First, we used only young adult faces (Figure 1). Earlier face recognition research, such as that reviewed by Rhodes and Anastasi (2012), suggests that the faces of one’s own age group are easier to remember than are those of another age group. This age-related difference regarding faces may have affected our results. A future study should use various faces from different age groups to ascertain whether the own-age bias influences gaze behavior. Second, we did not measure subjective emotional states, such as depression, in either the younger or older adults. Earlier studies have demonstrated that emotional factors affect decision-making processes (Miu et al., 2008). Because the older participants were recruited from a healthy population, people with severe depression were probably excluded. However, individual differences in depressive mood exist within the healthy population. Future studies must measure emotional states. Third, we used only neutral emotional stimuli for the decision-making tasks. An earlier study demonstrated that older adults devote more attention to emotionally positive stimuli (e.g., happy faces and positive words; Reed et al., 2014). Therefore, greater gaze bias in emotional decision-making may be observed in older adults. To verify this possibility, further research should use both emotional and neutral facial stimuli. Fourth, we asked only the younger adults to rate the attractiveness of the stimuli. Older adults may have different perceptions of attractiveness. Therefore, this difference may have affected our finding of age differences. Additionally, the temporal resolution of the eye tracker we used was limited to 60 Hz. This resolution is quite low compared to that of a previous study (Schotter et al., 2010) in which the eye tracker resolution was 1000 Hz. This difference in resolution may have affected our results. Moreover, we used only human faces as stimuli. Previous studies conducting a dwell duration analysis used several types of stimuli, such as landscapes or portraits (Glaholt and Reingold, 2009a,b; Schotter et al., 2010). Given that the human face captures more attention than do non-face objects (Bindemann et al., 2005), the gaze patterns observed during the first dwell time in this study would be expected to differ from those of previous studies. Although the underlying mechanisms remain unclear, this difference in stimuli may have affected our results. Finally, it is important to consider that the older adults in this study were healthy (aged 65–74 years). Therefore, additional studies of middle-aged or very old adults (75–90 years) should be performed to generalize the gaze bias effect. Additionally, studies of clinical populations or infants would help to generalize the gaze bias phenomenon and to elucidate the mechanisms thereof.

## Conclusion

In summary, we investigated whether gaze bias occurred in younger and older adults under different decision-making conditions. Our study provides the first scientific evidence that gaze bias occurs regardless of age, as only slight aging effects were observed for gaze behavior. The results demonstrate that a gaze bias for chosen stimuli can be generalized across age groups.

## Author Contributions

TS and HK designed and developed the study protocol and conducted the study. TS and RN analyzed data and wrote the manuscript. TS, RN, HK and RK provided critical revisions. All authors have read and approved the final manuscript.

## Conflict of Interest Statement

The authors declare that the research was conducted in the absence of any commercial or financial relationships that could be construed as a potential conflict of interest.

## Footnotes

^1^https://facegen.com/index.htm
