# Supplementary material for: Gaze Bias in Preference Judgments by Younger and Older Adults
Source: Front Aging Neurosci. 2017 Aug 25;9:285. doi: 10.3389/fnagi.2017.00285 (PMC5574931; doi:10.3389/fnagi.2017.00285)
Supplement: Supplementary file 1 [file Data_Sheet_1.docx]

Appendix 1. Average dwell duration values during first time and remaining time (s)

Note: Standard deviations are given in parentheses.
